# Improved structure of Zr-BTC metal organic framework using NH_2_ to enhance CO_2_ adsorption performance

**DOI:** 10.1038/s41598-023-44076-9

**Published:** 2023-10-17

**Authors:** Heidar Javdani Esfahani, Shahrokh Shahhosseini, Ahad Ghaemi

**Affiliations:** School of Chemical, Petroleum and Gas Engineering, Iran University of Science and Technology, Tehran, Iran

**Keywords:** Natural hazards, Engineering

## Abstract

Modified mesoporous NH_2_-Zr-BTC mixed ligand MOF nanocomposites were synthesized via the hydrothermal method as a novel adsorbent for CO_2_ capture. The newly modified MOF-808 with NH_2_ demonstrated a similar mesoporous morphology as MOF-808, whereas the specific surface area, pore volume, and average particle size, respectively, increased by 15%, 6%, and 46% compared to those of MOF-808. The characterization analyses exhibited the formation of more active groups on the adsorbent surface after modification. In addition, a laboratory adsorption setup was used to evaluate the effect of temperature, pressure, and NH_2_ content on the CO_2_ adsorption capacity in the range of 25–65 °C, 1–9 bar, and 0–20 wt%, respectively. An increase in pressure and a decrease in temperature enhanced the adsorption capacity. The highest equilibrium adsorption capacity of 369.11 mg/g was achieved at 25 °C, 9 bar, and 20 wt% NH_2_. By adding 20 wt% NH_2_, the maximum adsorption capacity calculated by the Langmuir model increased by about 4% compared to that of pure MOF-808. Moreover, Ritchie second-order and Sips models were the best-fitted models to predict the kinetics and isotherm data of CO_2_ adsorption capacity with the high correlation coefficient (*R*^2^ > 0.99) and AARE% of less than 0.1. The *ΔH*°, *ΔS*°, and *ΔG*° values were − 17.360 kJ/mol, − 0.028 kJ/mol K, and − 8.975 kJ/mol, respectively, demonstrating a spontaneous, exothermic, and physical adsorption process. Furthermore, the capacity of MH-20% sample decreased from 279.05 to 257.56 mg/g after 15 cycles, verifying excellent stability of the prepared mix-ligand MOF sorbent.

## Introduction

The climatic condition has worsened by daily emitted greenhouse gases (GHGs). Particularly, carbon dioxide is now a matter of ubiquitous public concern. The significant contribution to such a severe rise is associated with the combustion of the fossil fuels, including petroleum, natural gas, and coal^1^. These days, carbon dioxide capture and storage (CCS) is a practical approach to reduce greenhouse gas emissions^2, 3^.

Several techniques for capturing CO_2_ include chemical absorption, physical adsorption, and membrane separation processes. Amine-based chemical absorption has been widely used in the industry, primarily for CO_2_ capture^4–6^. Nevertheless, this technology needs a remarkable amount of energy to recover amines for recycling, and corrosion and toxicity drawbacks cause high cost and low energy efficiency^7, 8^. On the other hand, different kinds of sorbents can mitigate such problems^9^ because the adsorption technology can reduce the costs related to CO_2_ capture below the amine-based absorption process^10^. Commonly, activated carbons and zeolites have been utilized for gas removal and separation through adsorption, although their modification to improve selectivity is required^11, 12^.

As a type of porous, crystalline material, metal–organic frameworks (MOFs) are one of the fastest-growing fields in chemistry, chemical, and material science, which have encouraged many scientists to use them in multiple applications. Due to their high tunability in design, ultrahigh surface areas, and extensive structural and chemical diversity, they enjoy higher functionality with decreased adsorbent volume and mass compared to traditional adsorbents for different purposes, including gas removal/storage, heterogeneous catalysis, ion exchange, and molecular separation^13^. The existence of coordinatively unsaturated metal centers or open metal sites along the pore surfaces is the most attractive characteristic of these materials. Such five-coordinate metal cations are tunable to post-synthetic functionalization, which behave as Lewis acids to polarize gas adsorbents considerably. MOFs have been developed as promising alternative sorbents for CO_2_ capture thanks to their super-high porosity, regular porous architectures, and chemical functionalities which can be changed by adjusting the organic linker or metal group^14^.

There are various synthesis methods for MOFs: solvothermal, microwave-assisted, sonochemical, mechanochemical, electrochemical, sol–gel chemistry, seed-induced growth, continuous flow chemistry, and slow evaporation methods. The solvothermal approach is one of the most functional methods, which uses organic linkers, organic high-boiling solvent, and soluble metal salts in a sealed vessel and heated at a temperature higher than the boiling point of the solvent to perform the reaction. Then, the produced substance is recovered and washed, followed by solvent evacuation in MOF pores. This procedure allows to generate uniform MOF particles with high crystallinity, small crystallite size distribution, and high phase purity due to the fast reaction kinetics, although, some empirical optimization is still required^15^. In examining the process kinetics and diffusion barriers, specific surface area, average particle size, microporous volume, and size distribution are the crucial factors for various applications of MOFs involving mass transfer. For example, the mass transfer diffusion barrier is reduced with crystallite sizes in gas adsorption processes^16^.

Notwithstanding, one of the primary barriers to limiting the practical applications of MOFs is moisture stability since most of them are synthesized via weak metal–ligand coordination bonds, making them susceptible to water molecules in an atmospheric environment^17^. For example, although MOF-5 is extensively employed for different possible applications, it is vulnerable to water content. Moisture cannot be ignored in CO_2_ removal and environmental pollutant detection technologies^18^. The instability of MOFs towards moisture requires to be mitigated if they are exploited in the fields mentioned before. By virtue of their tailorable properties, their features and structures can be finely amended by a meticulous selection of building blocks, which may cause moisture-resistant MOFs, such as using ligands with high pKa quantity^19^. Generally, MOFs are made from metal ions or organic ligands as linkers and clusters as nodes, where the impact of the organic ligands can be predicted for their post-functional and designable natures^20^.

The particle size is one of the most significant physicochemical characteristics of MOFs, since it directly influences their intrinsic features. Therefore, adjusting the particle size can be a critical issue in different applications. In one procedure, additives are employed in the synthesis process to act as a growth inhibitor postponing the growth mechanism and decreasing the size. The characteristics of inhibitors are associated with the precursors as the competition between inhibitor and ligand in the reaction, resulting in decreased crystal growth and particle size of MOFs^21^. Another critical feature of MOFs is morphology, depending on different parameters. The versatility of organic ligands and metal nodes enables them to adjust the structures and compositions of MOFs. The organic ligands and metal ions with different optical, electronic, and magnetic characteristics could be selected for particular applications of MOFs^22^. The MOFs flexibility represents a capability of tuning morphology and size to maximize their porosity and surface area for diverse applications. The elongation of the ligand length and the incorporation of some additives into MOF structure are the methods for manipulating its pore size^23^. In recent decades, several researchers have reported the applications of different organic ligands to synthesize single and mix-ligand MOF-based adsorbents for CO_2_ capture, some of which are reported in Table 1.

**Table 1:** Summary of some recent studies on single and mix-ligand MOF-based sorbents for CO_2_ capture.

Author	Sorbent	Ligands	T (K)	P (bar)	Max. Adsorption Capacity (mg/g)	Synthesis strategy	References
Ye et al. 2013	HKUST-1	–	303–473	0–10	80.09	Teflon-lined steel autoclave	^24^
	MIL-101 (Cr)				51.49		
Yu et al. 2017	Multi-Cage-Based MOF	TPT and carboxylic acids	273–298	0.1–1	211.24	Reticular	^25^
Wang et al. 2015	Polyhedron-based MOF	Cu-paddlewheel and bent tetracarboxylate	273–298	0.1–1	318.19	Supermolecular building blocks (SBBs)	^26^
McDonald et al. 2012	Alkylamine appended MOF	mmen-Mg_2_ (dobpdc)	298–393	0–1.5	138.19	Solvothermal and microwave	^27^
Gao et al. 2018	Zn-based MOF	Expanded tricarboxylic acid	273–298	0–1	185.72	Solvothermal reaction	^28^
McDonald et al. 2011	MDEA incorporated in MOF	mmen-CuBTTri	298	0–1.5	104.74	Grafting	^29^
Mu et al. 2015	NbO-type MOF	Diisophthalate with a PZ-Ring Bridge	273–293	0.1–1	212.56	N-alkylation process	^30^
Qiu et al. 2020	Flexible–Robust Copper(II) MOF	Fluorinated	273–298	0.1–1	70.41	Trifluoromethyl-functionalized linear dicarboxylate	^31^
Zou et al. 2018	Zeolite-like MOF	tetrahedral organic	273–298	0–1	241.17	4 + 4 synthetic strategy	^32^
Maity et al. 2018	IISERP-MOF24	4,4′-biphenyldicarboxylate (BPDC) & imino diacetate (IMDA)	195–298	1	88.02	Solvothermal	^33^
Yuan et al. 2019	Copper cluster-based MOFs	isophthalate and tetrazolate	77–298	0–0.0013	172.08	Secondary building unit (SBU)	^34^
Parshamoni and Konar 2016	Zn(II) based MOFs	2,5-thiophene dicarboxylic acid, 2-Aminoterephthalic acid, 2 6-naphthalene dicarboxylic acid, adipic acid, N,N-bis-pyridin-4-ylmethylene-hydrazine	195–298	P/P_0_ = 0–1	63.81	Slow diffusion technique	^35^
Kang et al. 2020	N-oxide-functionalized Cu-based 3D flexible microporous MOF	2,20-bipyridyl-3,30-dicarboxylic acid-1,10-dioxide (H2bdd)	77–298	0–0.0013	–	–	^36^
Wang et al. 2014	Chiral flexible MOFs	2,2′-bipyridyl-5,5′-dicarboxylate (H2bpydc) and trinuclear clusters	195–298	0–1	74.81	–	^37^
Wang et al. 2018	Copper-based MOF	bent diisophthalate	77–298	0–1.06	172.08	Two-fold Suzuki–Miyaura cross-coupling reaction	^38^
Liu et al. 2016	NbO-type copper MOF	pentacarboxylate ligand and paddlewheel SBU	77–298	0–1.1	4.41	Solvothermal	^39^

Among the present MOF samples, studied in different engineering applications, the MOF-808 has attracted more attention for the CO_2_ capture purposes due to several factors. Firstly, MOF-808 exhibits a high gas adsorption capacity, particularly for CO_2_, owing to its large surface area and pore volume. Secondly, the material demonstrates excellent stability and compatibility, ensuring the retention of its structural integrity and adsorption capabilities during modification. Additionally, the straightforward synthesis methods of MOF-808 facilitated the introduction of amine functional groups, allowing for easy modification. The incorporation of amines enhances the selectivity for CO_2_ adsorption, given their strong affinity for CO_2_ molecules. Finally, the research addresses a novel aspect by exploring the interactions between MOFs and amines, particularly at different weight percentages, thereby contributing to the field of amine-modified MOFs^40^. Recently, mixed ligand MOFs have received much consideration in different separation and conversion applications. This work aims to introduce a novel adsorbent for CO_2_ capture process. Pre-synthesis modification of the MOF-808 are conducted through incorporation of an amine-based ligand into the MOF structure to manipulate its structural properties and morphology. To do so, Zr-BTC MOF was modified with an NH_2_ ligand at different concentrations to obtain a novel amine modified MOF-808 sample with high CO_2_ adsorption capability. In addition, the resulting samples’ elemental composition and morphological properties are investigated by utilizing FTIR, SEM, EDS, XRD, and BET analysis. Furthermore, due to the lack of a predictive model for CO_2_ uptake capability of the resulting MOF samples, the CO_2_ adsorption isotherm and kinetic modeling are applied to provide predictive models, which are necessary for CO_2_ capture plant design. In addition, the adsorption process feasibility are investigated by providing thermodynamic parameters, and also the CO_2_ gas diffusion coefficients are presented to study the effect of the MOF-808 amine functionalization on the CO_2_ mass transfer. Finally, the adsorbent recoverability is examined under successive cycles to ensure their stability for future applications.

## Experimental

### Materials

For preparing MOF-based adsorbents through the hydrothermal method, different materials were used without any modification, including 5-Aminoisophthalic acid (AIPA, > 98% purity), Zirconium (IV) oxide chloride octahydrate (ZrOCl_2_, 98% purity), 1,3,5-Benzenetricarboxylic acid (BTC, > 98% purity), Formic acid (98–100% purity), N, N-Dimethylformamide (DMF, > 99.9% purity), Chloroform (> 99% purity), and Acetone (> 99.5% purity). The first one was supplied by Sigma Aldrich Co., while the others were purchased from Merck Co.

### Synthesis method

#### MOF-808

**Figure 1:**
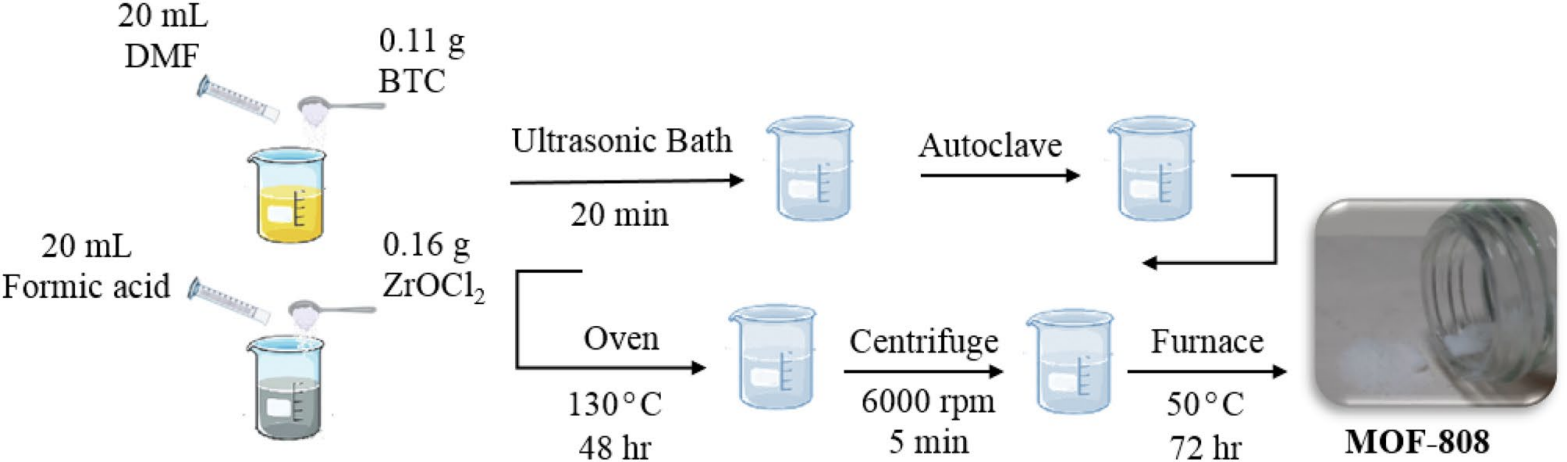
Synthesis procedure and steps of pure and improved Zr-BTC MOF by NH_2_.

In the first solution, 0.11 g BTC (0.5 mmol) was mixed with 20 mL DMF, while in the second solution, 0.16 g ZrOCl_2_ (0.5 mmol) was mixed with 20 mL formic acid. The solutions were mixed separately in an ultrasonic bath for 20 min to become entirely homogenous. Then, both solutions were poured into a container and placed in the ultrasonic bath for 20 min to be completely mixed. Next, the resultant mixture was added to the reactor (autoclave with a volume of 300 mL) and placed in an oven at 130 °C for 48 h. Then, the excess solvent from the synthesized solution was separated with a pasteurizer pipette, transferred to the falcon, washed three times with DMF (30 mL for 5 min) and three times with acetone (30 mL for 5 min), and then centrifuged at 6000 rpm for 5 min. After washing and centrifuging, the precipitate was washed with acetone and poured into the oven at 100 °C to dry completely. Then, the dried precipitate was separated, mixed with chloroform for 15 min on the heater under the hood until it became homogeneous and put in the furnace at 50 °C for 72 h. Finally, the excess solvent was removed, and the remaining material was placed in the oven at 100°C to dry and form MOF-808 (MH-0%). The scheme of the synthesis procedure of all prepared samples is depicted in Fig. 1.

#### MOF-808-%NH_2_

**Figure 2:**
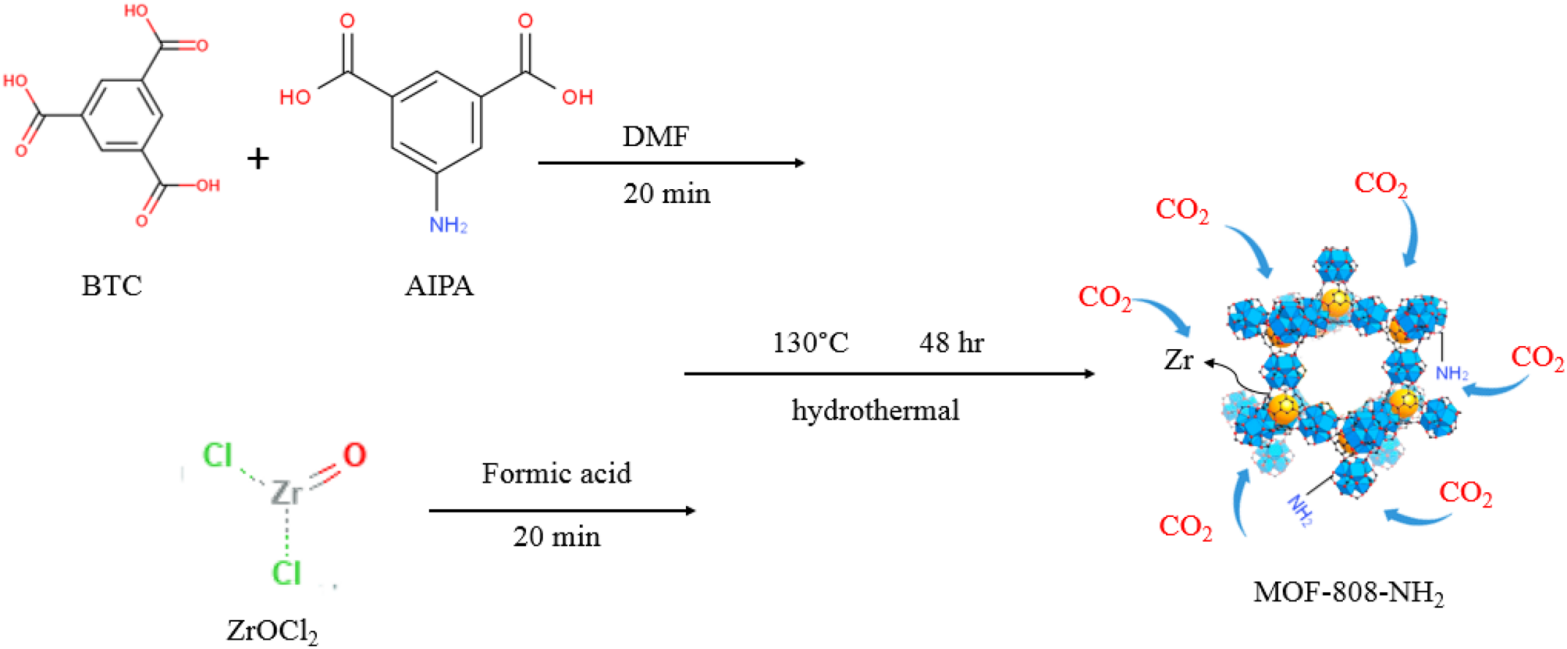
Molecular structure of MOF-808/NH_2_ nanocomposite and its CO_2_ adsorption mechanism.

Two mixed ligands MOF samples (solutions) were prepared using the following procedure. The first solution was prepared by mixing specific masses of BTC and AIPA (0.094 g: 0.009 g @MH-10% & 0.084 g: 0.018 g @MH-20%), and the second solution was a mixture of 10 mL acid formic and 0.16 g ZrOCl_2_. These solutions were kept in the ultrasonic batch for 20 min to homogenize completely. The ligands were first mixed, and then the metal was added to them while placed in the bath for 20 min to become homogenous. After this, a procedure similar to that of the previous section was followed to prepare MH-10% and MH-20%. The molecular structure of the improved MOF-808 samples is represented in Fig. 2.

### Material characterization

The synthesized adsorbents were characterized by various analyses as follows. The composition and crystalline structure of the samples and their phases were identified by X-ray diffraction (XRD, STOE STADI-MP, Germany) under Cu Ka radiation, 40 kV voltage, 30 Ma flow, and 2θ range of 4°–84°. The phase-detection was conducted by X'Pert HighScore Plus software. To characterize the quantity of the defective ligand in the final structure of the MOF samples, proton nuclear magnetic resonance (^1^H-NMR) spectroscopy was conducted at 600 MHz by utilizing a Brucker spectrometer device. To do so, the synthesized MOF samples were dissolved separately in 5 mL Deutero-hydrochloric acid solution (DCl-20%) contain 5 mg Cesium Fluoride (CsF) for 7 h, followed by adding Hexadeuterodimethyl sulfoxide (DMSO-d_6_) to the resulting solution. The mentioned digested samples were used for performing ^1^H-NMR analysis. The functional groups present in each sample structure were analyzed by Fourier Transform Infrared spectroscopy (FTIR, Spectrum Rx1, Perkin Elemer Company) analysis in 400–4000 cm^−1^. The samples were analyzed by Brunauer–Emmett–Teller (BET, Micromeritics, Model ASAP 2020, USA) under the temperature of 77K and a pressure range of 7–22 kPa after eliminating any possible impurities within the sample volume by heating at 100 °C. The mesopore surface area, pore diameter and volume of the samples were calculated by Joyner–Halenda (BJH) method. The adsorbent morphology and size were evaluated by the Field emission scanning electron microscopy (FESEM) technique, and their elemental composition was examined by energy dispersive spectroscopy (EDS) with the help of an S-4700 microscope (Hitachi, Japan). The resulting MOF samples particle size were measured through processing the FESEM images by using Image J software (National Institute of Health)^41^, also Dynamic Light Scattering (DLS) analysis was performed to measure particle size using particle size analyzer (zetasizer model, Malvern) at the scattering angle of 90°.

### Experimental setup

**Figure 3:**
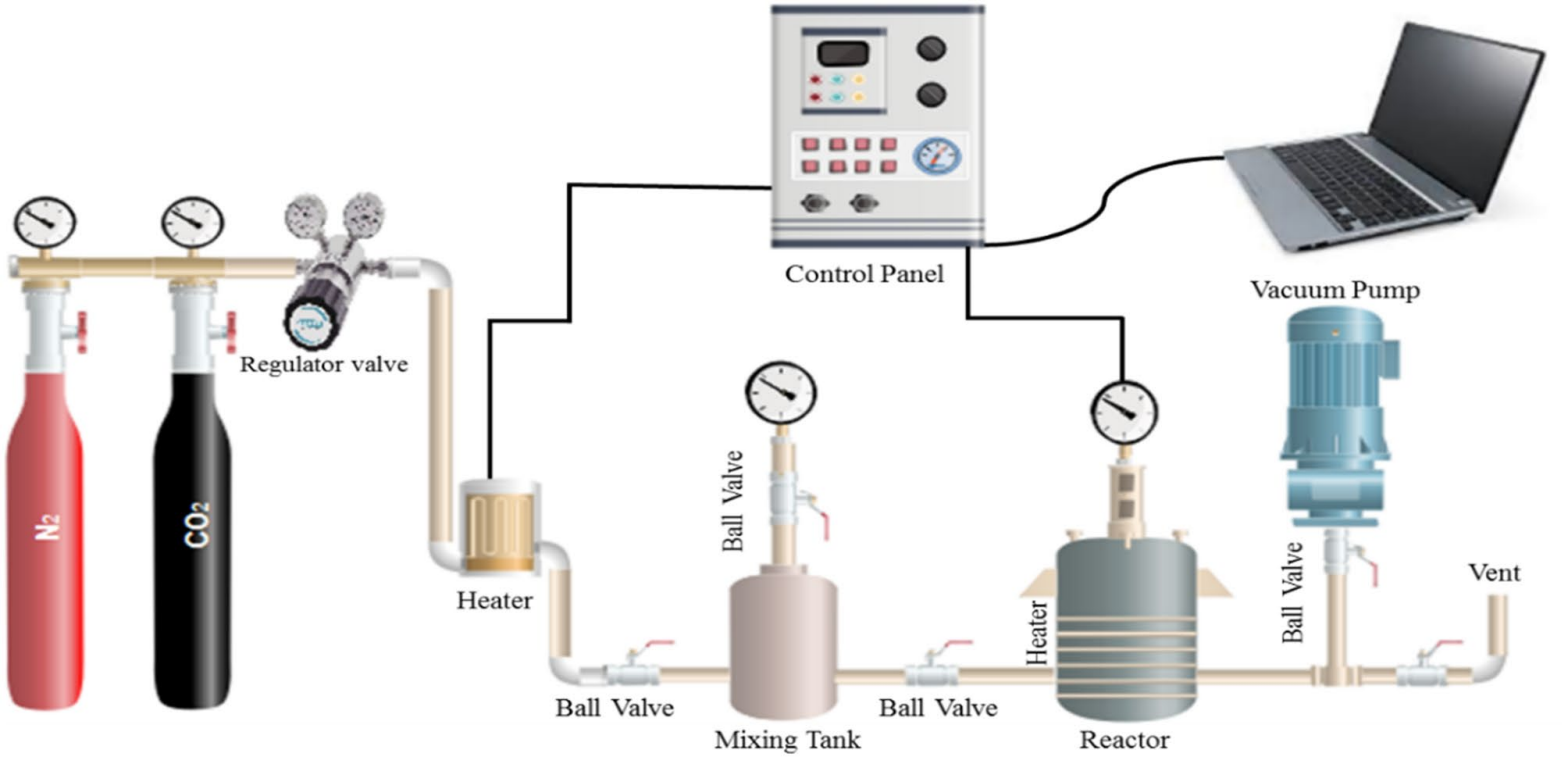
Scheme of experimental setup used for CO_2_ adsorption process.

A lab-scale batch stainless steel reactor was designed with an inner radius of 3 cm, a volume of 254 cm^3^, and a height of 9 cm. The reactor was entirely isolated with an appropriate sealed cap to minimize gas waste during the adsorption process. A cell was embedded in the reactor volume to place the adsorbent. The process pressure and temperature were adjusted at the desired values by a pressure gauge and a connected valve while displaying them online on a digital panel. A heater was attached between the regulator valve and the mixing tank to pre-heat the and achieve the desired feed temperature. To start the experiments, N_2_ gas entered the reactor to remove the air in it. Afterwards, the adsorbent (0.5 g) was weighed by a digital balance, poured into the cell, and then placed within the reactor chamber. Then, the valve was opened to allow CO_2_ gas to enter into the reactor until reaching the specified initial pressure. Since the adsorption data was almost fixed after 30 min, this period of time was considered the processing time. After finishing the process, the recorded results, including temperature change, pressure variation, and time, were employed to calculate the corresponding parameters. It should be stated that all the experimental runs were repeated four times, and the mean values were presented in this work. The current adsorption system includes a reactor, gas cylinder, valves, storage tank, and pressure gauge, as shown in Fig. 3.

### Experimental measurements

In the volumetric gas adsorption set up the amount of the adsorbed CO_2_ gas can be measured by calculating the differences in the mass of the CO_2_ gas at the initial adsorption time and the final adsorption time. The mass of the CO_2_ adsorbed throughout the adsorption process is calculated by the following equation:

1 $$q_{e} = \frac{{m_{i} - m_{f} }}{w} = \left( {\frac{VM}{{Rw}}} \right)\left( {\frac{{P_{i} }}{{Z_{i} T_{i} }} - \frac{{P_{f} }}{{Z_{f} T_{f} }}} \right)$$

where m_*i*_ and m_*f*_ are the mass of the CO_2_ gas inside the adsorption vessel at the initial adsorption time and the final adsorption time, respectively. The term *w* is the adsorbent weight, *V* is the reactor volume occupied by CO_2_ gas, *M* is CO_2_ gas molecular weight, *R* is the global gas constant, *P* is pressure; *T* is temperature, and* Z* is the compressibility factor determined by the Virial equation of state through the calculation of Virial coefficients using the Tsonopoulos correlations, as given below^42^.

2 $$Z = 1 + \frac{BP}{{RT}}$$

3 $$\frac{{BP_{c} }}{{RT_{c} }} = F^{(0)} (T_{R} ) + \omega F^{(1)} (T_{R} )$$

4 $$F^{(0)} (T_{R} ) = 0.1445 - \frac{0.330}{{T_{R} }} - \frac{0.1385}{{T_{R}^{2} }} - \frac{0.0121}{{T_{R}^{3} }} - \frac{0.000607}{{T_{R}^{8} }}$$

5 $$F^{(1)} (T_{R} ) = 0.10637 - \frac{0.331}{{T_{R}^{2} }} - \frac{0.423}{{T_{R}^{3} }} - \frac{0.008}{{T_{R}^{8} }}$$

where *B*, *P*_*c*_, *T*_*c*_, and *T*_*R*_ are the second Virial coefficient, gas critical pressure, critical temperature, and reduced temperature, respectively. The absolute average relative error (AARE%) and correlation coefficient (*R*^*2*^) are determined by the equations below^43^.

6 $$\% AARE = \left( {\sum\limits_{i = 1}^{N} {\left| {\frac{{q^{\exp } - q^{cal} }}{{q^{\exp } }}} \right|/N} } \right) \times 100$$

7 $$R^{2} = \frac{{(q^{\exp } - \overline{q}^{cal} )^{2} }}{{\sum\limits_{i = 1}^{N} {\left( {(q^{\exp } - \overline{q}^{cal} )^{2} + (q^{\exp } - q^{cal} )^{2} } \right)} }}$$

## Results and discussion

### Adsorbent characterization

**Figure 4:**
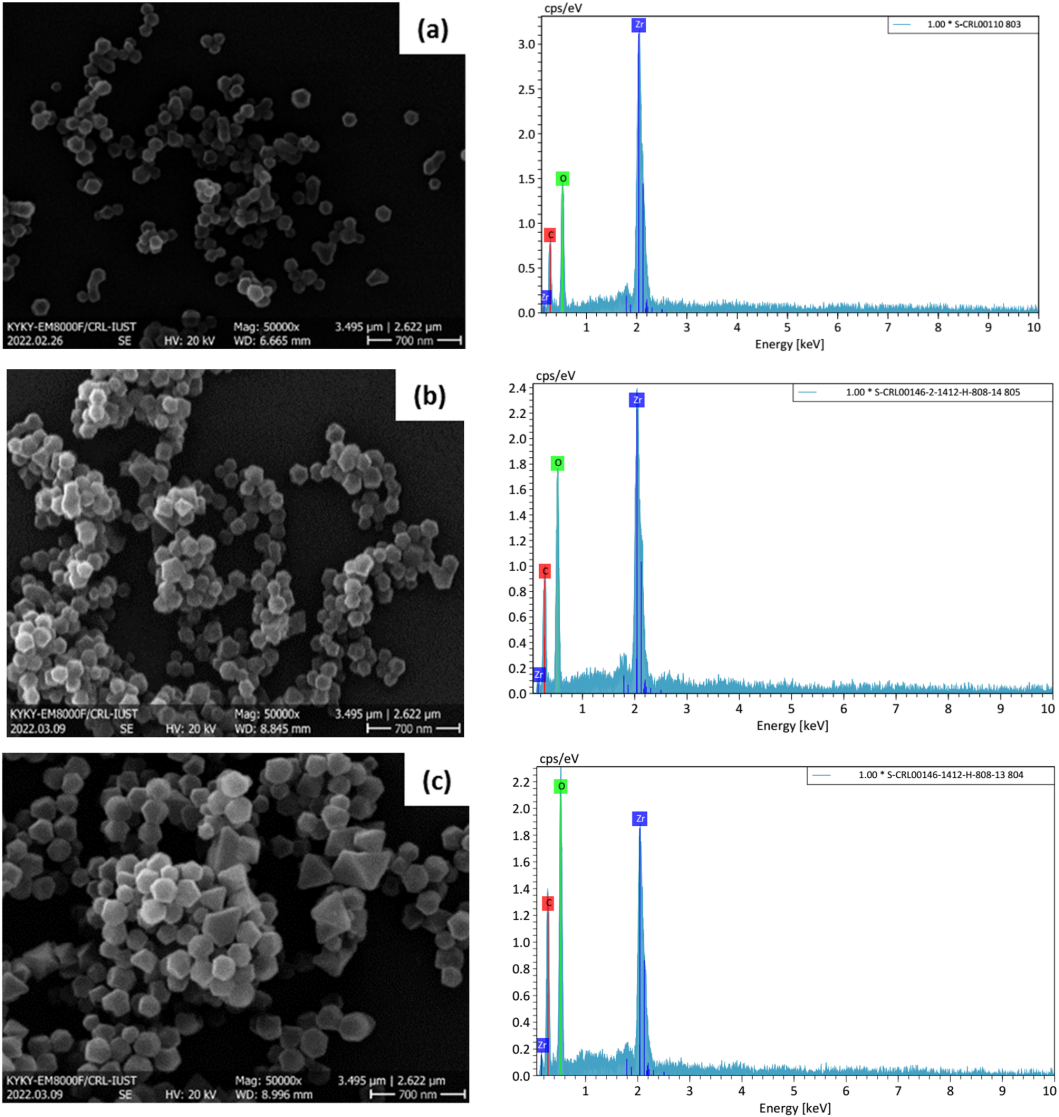
SEM images and EDS diagram of the synthesized samples; (**a**) MH-0%, (**b**) MH-10%, and (**c**) MH-20%.

**Figure 5:**
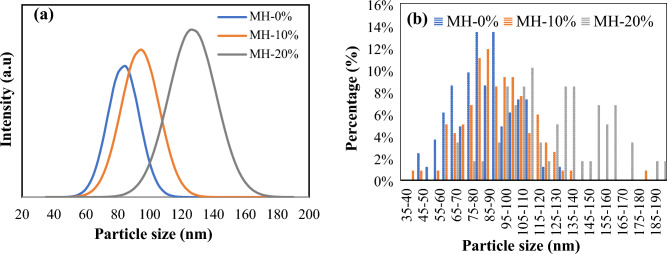
Particle size distribution of all adsorbents: MH-0%, MH-10%, and MH-20% (**a**) based on DLS analysis, and (**b**) based on image J software.

Visual analyses of the SEM images of the MOF-based adsorbents are represented in Fig. 4. The results indicate that typical octahedral microcrystals were formed in the MOF-808 structure, with a highly monodispersed and uniform particle distribution. Relatively homogeneous size distribution is detected for each synthesized sample, with the differences in their sizes, in which the particle size has been increased by adding NH_2_ (triangle shapes) into the MOF structure. However, the crystallinity of MOF-808 has not been destroyed, and the structure of MOF-containing NH_2_ samples remains almost intact. The elements C, O, and Zr are the main constituents within the synthesized samples, according to the results of the EDS analysis. Table 2 shows more NH_2_ content in the sample has increased the amount of N element in the modified MOFs. The minimum, the maximum, and the average particle sizes obtained from evaluating SEM images are reported in Table 3. Figure 5b depicts the distribution of particle size for different samples. By adding 20 wt% NH_2_, the average particle size has been increased from 85.86 nm for pure MOF-808 to 125.98 nm for MH-20%. Figure 5a represents particle size distribution of the MOF samples, obtained from DLS analysis. It indicates that 83.62 nm, 94.13 nm, and 126.85 nm are the average particle size values of MH-0%, MH-10%, and MH-20% samples, respectively. In general, the presence of a co-ligand with lower pKa value (higher acidity) in comparison to another ligand can improves the rate of nucleation and increase the coordination of the ligands to the metal nodes through gaining the rate of ligand deprotonation. The pKa value of the BTC are pKa_1_ = 2.86, pKa_2_ = 4.30, and pKa_3_ = 6.28 corresponding to three carboxylic acid moiety deprotonation, while the pKa value of the 5-AIPA is 3.69. Therefore, considering the order of pKa values of the mentioned ligands, it can be concluded that particle size improvement from 85.86 to 125.98 nm is related to the higher concentration of the 5-AIPA as a co-ligand, which can increase the rate of MOF nucleation and crystal growth^21, 44^.

**Table 2:** Elemental composition of all adsorbents obtained from EDS analysis.

Elements	Sample (%)		
	MH-0%	MH-10%	MH-20%
C	37.97	40.89	40.94
O	26.38	29.53	33.03
Zr	35.65	27.64	23.15
N	0	1.94	2.88

**Table 3:** Minimum, maximum, and average particle size of the adsorbents.

Sample	Min particle size (nm)	Max particle size (nm)	Average particle size (nm)
MH-0%	48.80	131.68	85.86
MH-10%	43.06	181.93	93.16
MH-20%	66.46	191.53	125.98

**Figure 6:**
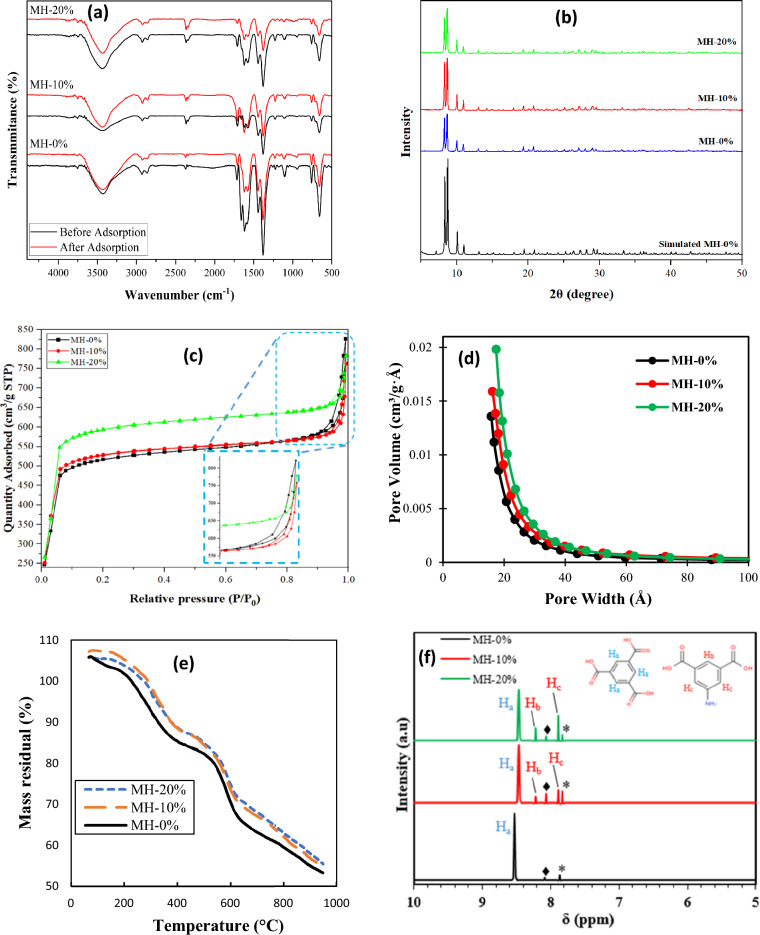
(**a**) FTIR spectra before and after adsorption process, (**b**) XRD patterns, (**c**) N_2_ adsorption/desorption isotherms, (**d**) Pore size distribution plots based on BJH method, (**e**) TGA results of all synthesized adsorbents, and (**f**) NMR spectra of the synthesized adsorbents (the symbols * and ♦ refer to the NMR peaks of the DMF and formic acid components).

FTIR spectra of the synthesized adsorbents, including MH-0%, MH-10%, and MH-20%, are displayed in Fig. 6a. The representative bands detected at 659 and 757 cm^−1^ in the spectra of all the sample composites are attributed to Zr–O vibration, and the band at 1576 cm^−1^ is related to the C–O=H vibration of the carboxyl group^45^. The scissoring vibration and bending vibration of N–H appeared at 1430 and 1575 cm^-1^ in the modified samples, which indicates that the amino group is a component of these samples^46^. Figure 6b depicts the XRD patterns of the MOF-808-based samples. The diffraction peak at 2θ of 4.34° is related to the (111) plane of MOF-808. The peaks at 2θ values of 8.32° and 8.69° are attributed to diffraction from the planes (311) and (222) of MOF-808, respectively, which is similar to the reports in the literature^47^. The diffraction results showed broad Bragg reflections, proposing the existence of the crystals with small sizes, as agreed with the SEM images. As shown, the diffraction peaks related to all the samples are almost the same, since addition of NH_2_ could not form any new crystal form in the sample^46^, suggesting that the structure of MOF is preserved after the modification. Nitrogen adsorption/desorption isotherms of all synthesized adsorbents were determined in order to calculate their porous parameters in 0.01 < *P/P*_0_ < 1.0. Figure 6c illustrates N_2_ adsorption–desorption isotherms related to all of the synthesized samples, also the Fig. 6c shows an apparent hysteretic loop for all of the samples related to the capillary condensation in the mesopores, supported by the typical type IV isotherm based on the IUPAC classification^48^. According to the Fig. 6c, the sharp increase in N_2_ adsorption at the relative pressure (*P/P*_0_) lower than 0.05 is attributed to the presence of the micro pores in the MOF sample structure. The presence of hysteresis loop at the higher relative pressure (*P/P*_0_ > *0.8*) can also be related to the large pores or intraparticle cavities in solid sorbents structure^49^. The surface area of the resulting MOF samples including MH-0%, MH-10%, and MH-20% and their related porous properties are summarized in Table 4. According to Table 4, the BET surface area of the MOF-808 has been increased from 1756 to 2021 m^2^/g by incorporation of 5-AIPA up to 20 wt%. In general, increasing the molecular length of the organic ligand used as the modification agent in the mixed ligand MOFs synthesis can be increased, resulting to more MOF surface area, pore volume, and average pore width^50^. Therefore, the surface area enhancement of MOF-808 can be related to the greater molecular length of the 5-AIPA (6.9 Å), as the modification agent, compared to the BTC ligand. In addition, Fig. 6d indicates increasing the pore volume of the MOF from 0.943 m^3^/g for MH-0% to 1.043 m^3^/g for MH-20% and gaining the pore width of the NH_2_ modified MOF sample^51^. The ^1^H-NMR spectra of the MOF samples including MH-0%, MH-10%, and MH-20% are illustrated in Fig. 6f. In this figure the observed peaks allocated around 7.82 ppm and 8.08 ppm, indicated by * and ♦ symbols, are attributed to the DMF and formic acid presence in the MOF samples, respectively. According to the MH-0% sample’s spectra, a sharp peak at 8.54 ppm is correspond to the aromatic ring’s proton of the BTC linker. Considering the MH-10% and MH-20% samples spectra, two peaks appeared at 7.89 ppm and 8.22 ppm are related to the non-acidic or non-basic hydrogen of the 5-AIPA linker. By calculating the signal integration of the peaks, the quantity of the formic acid in the all samples and the 5-AIPA linker in the MH-10% and MH-20% samples were measured. Based on the results of the integration, the mole fraction of the formic acid in the MH-0%, MH-10%, and MH-20% were obtained around to be 0.78%, 2.64%, and 1.35%, respectively. Also, the mole fraction of the 5-AIPA linker (mole of 5-AIPA/mole of 5-AIPA + BTC) in the MH-10% and MH-20% samples were obtained about 9.84% and 19.61% which are in acceptable agreement with the amount of linkers used during synthesis procedure^52, 53^. The TGA results of all MOF-based adsorbents are reported in Fig. 6e. The curves of the sorbents exhibited three-steps of mass losses. The first one with a loss of about 7% happened from 110 to 240 °C and is associated with the elimination of free solvent and moisture within the pores^54^. The second loss of approximately 20% occurred in 240–450 °C, which is attributed to the elimination of un-coordinated linkers and coordinated solvent, owing to the robust chemical bonding. The final weight loss of about 45% is related to the structural collapse of the MOF sorbents that has taken place at about 620 °C.

**Table 4:** Porous properties of the MOF-808 samples.

Sample	SA^a^_BET_ (m^2^/g)	MPV^b^ (m^3^/g)	PV^c^ (m^3^/g)	avg pore width^d^ (Å)
MH-0%	1756	0.601	0.943	20.72
MH-10%	1792	0.636	0.985	21.04
MH-20%	2021	0.705	1.043	22.43

^a^Specific surface area calculated by BET method.

^b^Micropore volume.

^c^Total pore volume.

^d^Average pore width calculated based on equation 4V/A by BET.

### The effect of operating conditions

**Figure 7:**
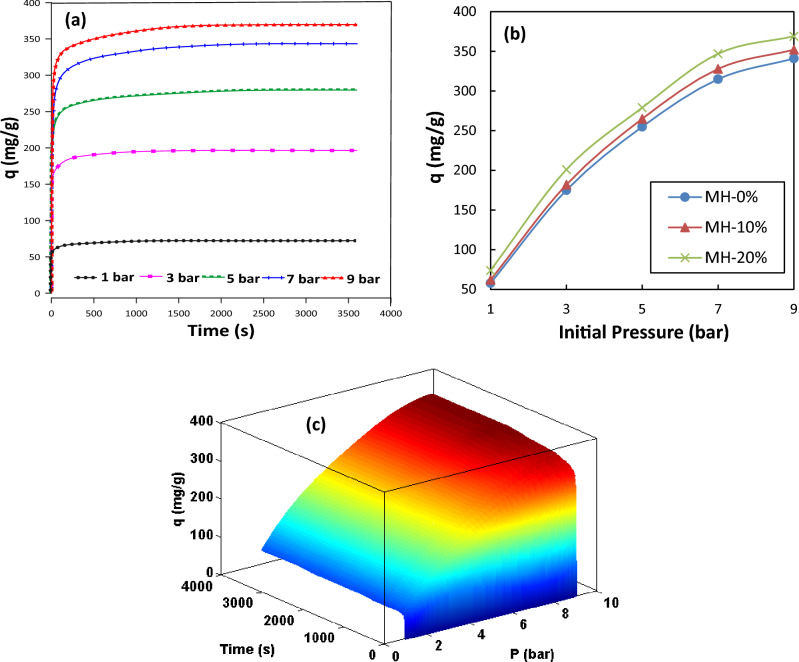
Variation of CO_2_ adsorption capacity (**a**) pressure effect versus time (2D), (**b**) types of the samples versus pressure effect, (**c**) pressure effect versus time (3D) at 25 °C for MH 20%.

**Figure 8:**
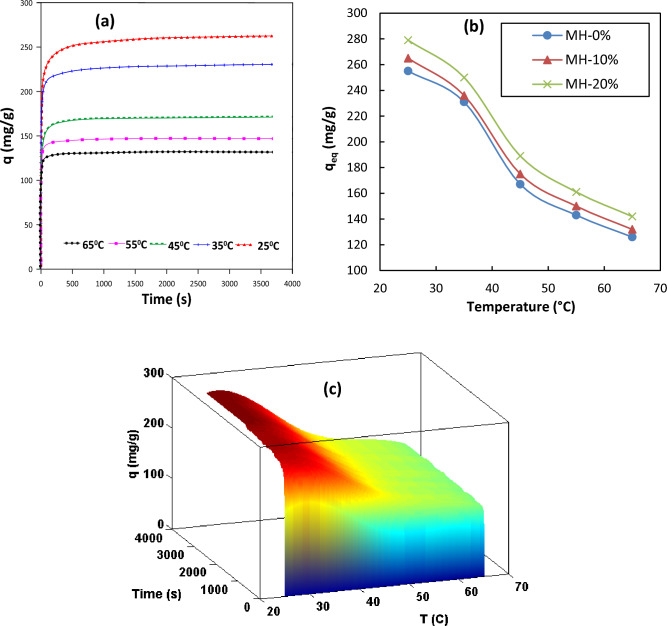
Variation of CO_2_ adsorption capacity (**a**) temperature effect versus time (2D), (**b**) types of samples versus temperature effect, (**c**) temperature effect versus time (3D) at 5 bar for MH 20%.

For all three synthesized MOF-based samples, the effects of two key operating conditions (process temperature and pressure) on the equilibrium and transient adsorption capacity data were examined. The results indicated that generally, the modified MOF sample with the maximum concentration of NH_2_ (i.e., MH-20% in the current study) performed better than the unmodified MOF-808 adsorbent. The MH-20% adsorbent exhibited the best adsorption performance. Figure 7 shows variation of adsorption capacity versus processing time (2D and 3D plots) under different operating pressures. As shown, the higher the process pressure, the higher the amount of adsorbed CO_2_ by adsorbents, proving the exothermicity of the adsorption process. On the other hand, according to Fig. 8, the capacity variation at different initial temperatures demonstrated a decrease in the amount of adsorbed CO_2_ by increasing temperature. This decline is a result of the breakdown of the weak van der Waals bands between the adsorbed gas and the adsorbent that occur at higher temperatures. Overall, in the range of the studied conditions, pressure had a greater impact on the adsorption capacity than temperature^49^.

### Adsorption kinetics

Kinetic models can have impacts on the reactor design and performance. Power-law correlations or more complicated functions can represent the reaction rate. Typically, almost all gas adsorption kinetic models are complicated because they depend on porous material chemical and physical characteristics. The kinetic models can readily express the interactions between sorbate and sorbent as well as sorbent behavior. The common kinetic models employed for CO_2_ adsorption on nanoparticles are the first-order, second-order, Elovich, and rate-controlling models. For example, the equilibrium reversibility of the adsorbent-adsorbate capture process can be represented by the first-order model, or the second-order model can propose the control hypothesis for the chemical adsorption reaction^55^.

The CO_2_ adsorption kinetic modeling was conducted using the theoretical kinetic models including first order, second order, Ritchie second order, rate controlling, and Elovich models, that are presented in Eqs. (8), (9), (10), (11), and (12), respectively^56^.

8 $${\text{First order}}: \;\;q_{t} = q_{e} \left( {1 - e^{{k_{1} t}} } \right)$$

9 $${\text{Second order}}: \;\;q_{t} = \left( {q_{e}^{2} k_{2} t} \right)/\left( { 1 + q_{e} k_{2} t} \right)$$

10 $${\text{Ritchie second order}}: \;\;q_{t} = q_{e} - q_{e} /\left( { 1 + k_{3} t} \right)$$

11 $${\text{Rate controlling}}: \;\;q_{t} = k_{c} t^{0.5}$$

12 $${\text{Elovich}}: \;\;q_{t} = \beta \ln \left( {\alpha \beta } \right) + \beta {\text{ln}}\left( t \right)$$

**Figure 9:**
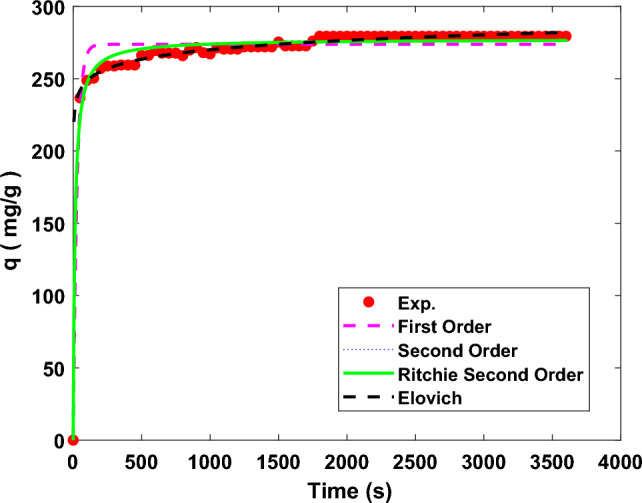
Kinetics of the adsorption process; adsorption capacity versus time at 25 °C and 5 bar for MH-20% adsorbent.

The kinetic data of the CO_2_ adsorption process for all of the adsorbents at 25 °C and various process pressures, as reported in Table 5. The kinetic plots are represented in Fig. 9 at a pressure of 5 bar to recognize the trend of adsorption capacity versus time. Although the data related to the rate-controlling model is reported in Table 5, its curve is not shown in the figure because of its lower *R*^2^ value compared to others. Similar trends are observed for all of the adsorbents, with a difference in the amount of adsorbed CO_2_. As mentioned before, the MH-20% sample was a more efficient adsorbent than others to adsorb CO_2_ under the studied operational conditions.

**Table 5:** Detailed data of kinetics models for CO_2_ adsorption at 25 °C and different operating pressures.

Model	Sample	MH-0%					MH-10%					MH-20%				
	P (bar)	1	3	5	7	9	1	3	5	7	9	1	3	5	7	9
First order	*q*_*e*_ (mg/g)	50.432	165.52	248.39	309.52	332.21	58.07	173.83	261.21	323.29	338.31	70.41	195.61	273.75	336.04	363.27
	*k*_*l*_	0.0096	0.0334	0.0322	0.0381	0.0402	0.0329	0.0393	0.0343	0.0395	0.0329	0.0428	0.0425	0.0361	0.0319	0.0363
	*R*^2^	0.794	0.950	0.977	0.983	0.980	0.954	0.974	0.981	0.984	0.960	0.962	0.978	0.975	0.972	0.977
Second order	*q*_*e*_ (mg/g)	53.30	168.39	251.98	313.01	335.97	58.95	175.95	264.53	326.73	334.00	71.19	197.68	277.35	341.27	368.04
	*k*_2_	2.6E−4	3.6E−4	2.8E−4	3E−4	2.8E−4	0.0011	5E−4	3.1E−4	3.1E−4	1.8E−4	0.0014	5.2E−4	2.9E−4	1.9E−4	2.2E−4
	*R*^2^	0.895	0.976	0.994	0.995	0.992	0.972	0.988	0.994	0.995	0.984	0.975	0.990	0.991	0.992	0.993
Ritchie second order	*q*_*e*_ (mg/g)	53.30	168.39	251.98	313.01	335.97	58.95	175.95	264.53	326.73	344.01	71.19	197.68	277.36	341.27	368.04
	*k*_*3*_	0.0136	0.0607	0.7006	0.0935	0.0953	0.0689	0.0885	3.1E−4	0.0997	0.0622	0.0996	0.1030	0.08007	0.0658	0.0803
	*R*^2^	0.895	0.976	0.994	0.995	0.992	0.972	0.988	0.994	0.995	0.984	0.975	0.990	0.991	0.991	0.993
Elovich	*α*	0.734	1.8E5	2.7E8	2E11	2E10	5.9E7	1.8E9	3.4E9	4.3E11	4.4E5	5.4E9	5.5E10	3.1E8	1.6E7	9.6E8
	*β*	5.708	7.710	8.590	8.711	9.963	2.232	5.721	8.317	8.909	14.714	2.306	5.788	9.400	12.701	11.921
	*R*^2^	0.934	0.974	0.949	0.954	0.975	0.814	0.966	0.954	0.973	0.992	0.842	0.969	0.975	0.958	0.967
Rate controlling	*k*_*id*_	1.135	3.705	5.532	6.889	7.402	1.305	3.877	5.817	7.196	7.562	1.572	4.36	6.104	0.7491	8.094
	*R*^2^	0.860	0.619	0.526	0.494	0.513	0.550	0.535	0.517	0.495	0.596	0.542	0.517	0.539	0.547	0.523

The empirical data do not entirely fit the first-order kinetic model, so it is not suitable to estimate the kinetics data, since the adsorption process is neither a simple chemisorption nor a simple physisorption. According to the hypothesis, if the controlling factor is chemisorption in adsorption processes, the kinetics corresponds to the second-order model. The rate-controlling kinetic model exhibits both types of chemical and physical adsorption. The results also indicate that the CO_2_ chemical adsorption happens on the active sites of the nanoparticles, where the adsorption rate is supposed to be directly proportional to the driving force (*n*th power) and the adsorption time (*m*th power). In all cases, the second-order and Ritchie second-order kinetic models are the best-fitted models with *R*^*2*^ values higher than 0.97, except for MH-10% at 1 bar, in which the Elovich model predicts the experimental data better than others.

### Adsorption isotherms

Isotherm models can present detailed information about the mechanism of the adsorption process, which is critical to design adsorption systems. Such models describe the type of reaction and interaction between adsorbents and adsorbates in gas capture processes. The isotherm models applied in the current study are Langmuir, Freundlich, Dubbin–Radushkevich (D–R), Temkin as two-parameter models, and Sips as a three-parameter model to describe the adsorption process^57^. The mentioned models are represented in Eqs. (13), (14), (15), (16), and (17), respectively.

13 $${\text{Langmuir}}:\;\;q_{e} = \frac{{q_{m} K_{l} P_{e} }}{{1 + K_{l} P_{e} }}$$

14 $${\text{Freundlich}}:\;\;q_{e} = k_{F} P_{e}^{{{\raise0.7ex\hbox{$1$} \!\mathord{\left/ {\vphantom {1 n}}\right.\kern-0pt} \!\lower0.7ex\hbox{$n$}}}}$$

15 $${\text{Dubinin}}{-}{\text{Radushkevich}}:\;\;q_{e} = q_{s} {\text{exp}}\left( { - k_{ad} \times E_{a}^{2} } \right)$$

16 $${\text{Temkin}}:\;\;q_{e} = B Ln A + B Ln P_{e}$$

17 $${\text{Sips}}:\;\;q_{e} = \frac{{ k_{s} \beta_{s} P_{e}^{{a_{s} }} }}{{1 + \beta_{s} P_{e}^{{a_{s} }} }}$$

**Figure 10:**
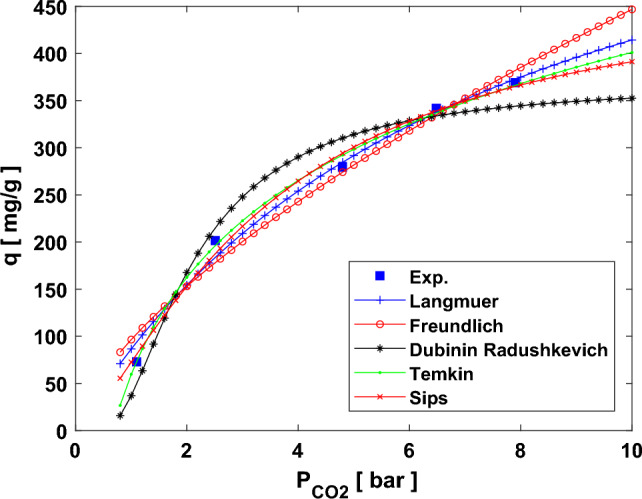
Isotherm plots of the adsorption process; equilibrium adsorption capacity versus final pressure at 25 °C for MH-20% adsorbent.

Figure 10 displays the CO_2_ adsorption curves at a temperature of 25 °C and various initial pressures in 1–9 bar. Since the adsorption tendency can be recognized by the constants of Langmuir and Freundlich isotherm models, the reduction in such constants can represent the physisorption behavior of CO_2_ capture using the synthesized adsorbents by adding NH_2_, as reported in Table 6. On the other hand, the change in *q*_*m*_ values in these models indicates that pressure and temperature considerably influence CO_2_ adsorption performance. An increase in the temperature and a decrease in the temperature caused a reduced *q*_*m*_ value due to the exothermic process. In the Freundlich model, it is assumed that the occupancy of more robust relating sites is prioritized, and then other sites with the process of decreasing energy, demonstrating the heterogeneous adsorbent surface and the multilayer adsorption caused by the nonhomogeneous energy distribution within the active sites. This occurrence in the Freundlich model presents the physical adsorption process. In contrast, the Langmuir model can describe the chemical reaction owing to an individual layer adsorption mechanism. The constant *n* is the Freundlich model. It can prove the desirability of adsorption if its value is in the range of 1–2. In addition, the term *E* and *B* in the D–R and Temkin models represent the free energy of adsorption and the adsorption heat, respectively^58^. If the value of *E* is less than 8 it indicates the physical adsorption, while its value in the range of 8–16 shows the chemical adsorption. The CO_2_ adsorption mechanism by all three adsorbents is a physical since their E values are less than 1^59^. The nonlinear regression method is used to calculate *R*^2^ values to find the best model, as well as check the AARE% as the second criterion. The best-fitted model had the highest *R*^2^ and lowest AARE quantities. With this in mind, although the Temkin model was the best in terms of *R*^2^ value (higher than 0.99 for all samples), the Sips model was selected as the most appropriate model for the estimation of isotherm data considering both criteria (*R*^2^ value higher than 0.99 and AARE% less than 0.3%). Considering both criteria, the order of the isotherm models' strengths to predict and to describe the adsorption behavior is as follows: Sips > Langmuir > Freundlich > Dubinin-Radushkevich > Temkin.

**Table 6:** Detailed data of isotherm models of CO_2_ adsorption process at 25 °C for all three adsorbents.

Model	Parameter	Sample		
		MH-0%	MH-10%	MH-20%
Langmuir	*q*_*m*_ (mg/g)	690.721	695.765	715.164
	*k*_*l*_	0.124	0.132	0.138
	*R*^2^	0.993	0.992	0.993
	*AARE%*	0.416	0.449	0.346
Freundlich	*k*_*f*_	96.501	90.551	85.896
	*n*	1.502	1.484	1.472
	*R*^2^	0.986	0.982	0.984
	*AARE%*	0.5983	0.688	0.573
Dubinin-Radushkevich	*q*_*s*_	368.253	365.622	351.232
	*k*_*ad*_	0.806	0.924	1.081
	*E*_*a*_	0.787	0.735	0.680
	*R*^2^	0.981	0.980	0.979
	*AARE%*	0.592	0.951	1.068
Temkin	*A*	1.496	1.580	1.606
	*B*	148.244	37.271	130.388
	*R*^2^	0.998	0.993	0.991
	*AARE%*	6.669	6.367	6.382
Sips	*K*_*s*_	85.501	76.188	74.110
	*β*_*s*_	1.392	1.429	1.317
	*a*_*s*_	0.178	0.166	0.153
	*R*^2^	0.996	0.995	0.995
	*AARE%*	0.248	0.153	0.139

### Adsorption thermodynamic

Van’t Hoff equations can be utilized to measure the thermodynamic parameters, including reaction enthalpy difference (ΔH°), entropy difference of sorbent and adsorbate interactions (ΔS°), and Gibbs free energy (ΔG°) for which the equations are given below^60^.

18 $$\ln K_{d} = \frac{\Delta S^\circ }{R} - \frac{\Delta H^\circ }{{RT}}$$

19 $$K_{d} = (P_{i} - P_{e} )\frac{V}{m}$$

20 $$\Delta G^\circ = \Delta H^\circ - T\Delta S^\circ$$

where *T* is temperature, *P*_*i*_ is the initial pressure, *P*_*e*_ is the equilibrium pressure, *K*_*d*_ is distribution factor, *V* is the volume, *m* is the mass, and *R* is the gas constant. A negative *ΔH*° quantity indicates an exothermic adsorption process, while a positive one demonstrates an endothermic one. Negative quantities of *ΔS*° show a high number of adsorbate molecules during the adsorption process. Entropy reduction throughout the process is due to a lower degree of freedom of the gas molecules, leading to the minimum free space on the carbon surface.

**Figure 11:**
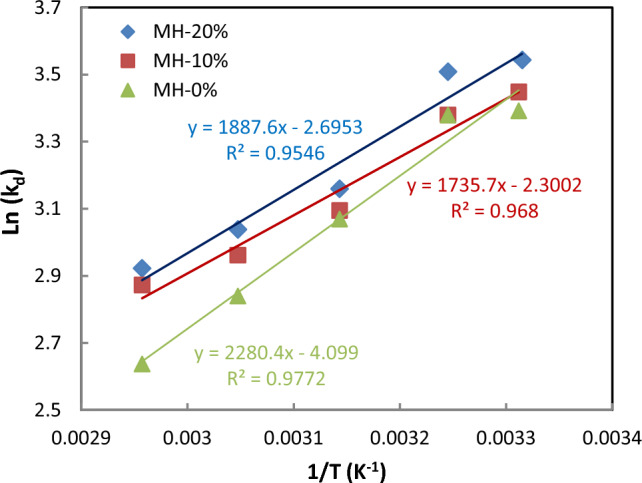
Van't Hoff plot for CO_2_ adsorption by all synthesized adsorbents at 9 bar.

The thermodynamic investigation of the physical and chemical adsorption processes can characterize the adsorption mechanism and the interaction between adsorbate and adsorbent. The thermodynamic parameters are calculated based on the Van’t Hoff equations. Figure 11 shows the Van’t Hoff plot of the equilibrium constant of the adsorbents in the temperature range of 25–55 °C. This plot is used to compute the enthalpy and entropy of the CO_2_ adsorption reaction. Moreover, Table 7 presents the calculated thermodynamic parameters for the adsorption process. The absolute amount of *ΔH*_0_ below 20 kJ/mol represents absolute physical adsorption, while above 40 kJ/mol expresses absolute chemical adsorption^61^. According to the parameters reported in Table 7, CO_2_ adsorptions by pure MOF-808 and NH_2-_modified are exothermic and spontaneous, with a feasible physical mechanism indicating less randomness of the adsorbent at the solid–gas interface throughout the adsorption process.

**Table 7:** Thermodynamic parameters of CO_2_ adsorption process on all synthesized adsorbents at 5 bar.

Sample	ΔH (kJ/mol)	ΔS (kJ/mol K)	ΔG (kJ/mol)				
			25 °C	35 °C	45 °C	55 °C	65 °C
MH-0%	− 18.615	− 0.033	− 8.661	− 8.457	− 8.127	− 7.797	− 7.747
MH-10%	− 16.327	− 0025	− 8.703	− 8.547	− 8.294	− 8.041	− 7.790
MH-20%	− 17.360	− 0.028	− 8.975	− 8.764	− 8.516	− 8.238	− 7.960

### CO_2_ diffusion coefficient calculation

**Figure 12:**
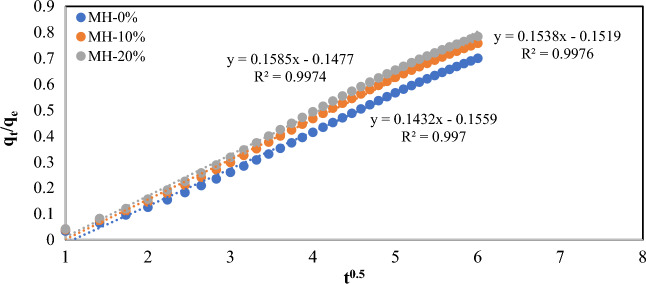
Plot of ($${\raise0.7ex\hbox{${q_{t} }$} \!\mathord{\left/ {\vphantom {{q_{t} } {q_{e} }}}\right.\kern-0pt} \!\lower0.7ex\hbox{${q_{e} }$}}$$) versus the square root of time (t^0.5^).

In order to investigate the effect of particle size on the CO_2_ gas diffusion into the resulting MOF samples (bulk powder), the CO_2_ diffusion coefficients were calculated for the modified/unmodified MOF samples similar to the study conducted by Zhao et al.^62^. Diffusion coefficients were calculated using Eq. (21), which is represented below:

21 $${\raise0.7ex\hbox{${q_{t} }$} \!\mathord{\left/ {\vphantom {{q_{t} } {q_{e} }}}\right.\kern-0pt} \!\lower0.7ex\hbox{${q_{e} }$}} = \frac{6}{{r_{c} }}\sqrt {\frac{{D_{m} t}}{\pi }}$$

where q_t_, q_e_, r_c_, t_,_ and π refer to the adsorption capacity at the time t, equilibrium adsorption capacity at the infinity time, average adsorbent particle radius, time, and a constant number equal to 3.1415, respectively. The term D_m_ also refers to the average diffusion coefficient of CO_2_ molecule between 0 to a given adsorption time of t. It should be noted that the mentioned equation is applicable when the adsorption capacities ratio ($${\raise0.7ex\hbox{${q_{t} }$} \!\mathord{\left/ {\vphantom {{q_{t} } {q_{e} }}}\right.\kern-0pt} \!\lower0.7ex\hbox{${q_{e} }$}}$$) are lower than 0.7^62^. Figure 12 illustrate the plot of adsorption capacity ratio ($${\raise0.7ex\hbox{${q_{t} }$} \!\mathord{\left/ {\vphantom {{q_{t} } {q_{e} }}}\right.\kern-0pt} \!\lower0.7ex\hbox{${q_{e} }$}}$$) versus the square root of time (t^0.5^) for all of the samples. In this figure the adsorption capacity ratio data are fitted on a linear equation and the average diffusion coefficient can be calculated from the slope of the fitted line using Eq. (22). The result of diffusion coefficient calculation are summarized in Table 8.

22 $$D_{m} = \frac{{slope^{2} r_{c}^{2} }}{36 \pi }$$

**Table 8:** The results of CO_2_ diffusion coefficient (D_m_) calculation.

Sample	Avg particle size (nm)	Slope	D_m_ (cm^2^/s)	R^2^
MH-0%	85.86	0.1432	1.32 × 10^–13^	0.997
MH-10%	93.16	0.1538	1.79 × 10^–13^	0.997
MH-20%	126.98	0.1585	3.53 × 10^–13^	0.997

According to Table 8, the average diffusion coefficient (D_m_) is increased by increasing the particle size of the MOF sample from 1.32 × 10^–13^ (cm^2^/s) for MH-0% to 3.53 × 10^–13^ (cm^2^/s) for MH-20% sample. The average D_m_ enhancement can be related to the greater particle size of the MH-20%, which reduces the barrier of CO_2_ mass transfer into the solid sorbent bulk powder^62^.

### Adsorbent regenerability

**Figure 13:**
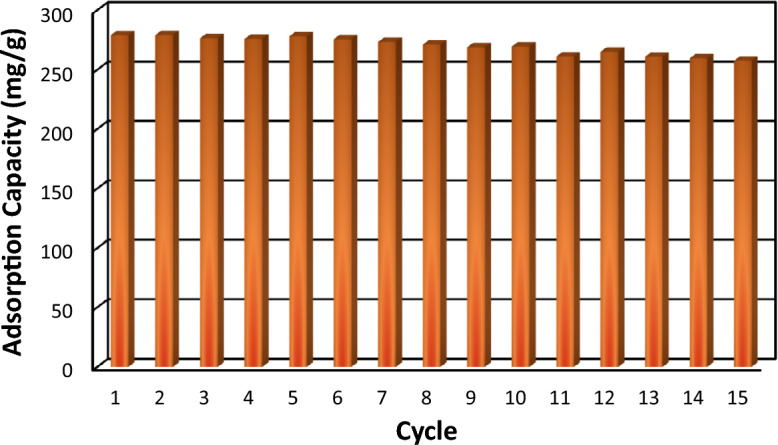
Regenerability of MH-20% sample for consecutive CO_2_ adsorption process.

The stability and regenerability of MOF-based adsorbents in the adsorption process are the important features that enhance the capacity and efficiency of CO_2_ capture. To investigate the regenerability of the adsorbent, several cycles of CO_2_ adsorption–desorption were conducted for 0.5 g adsorbent, using the mentioned experimental set up. The CO_2_ adsorption process was conducted at 25 °C and 5 bar for MH-20% sample, while the desorption process was conducted at 100 °C and 10^–5^ bar vacuum for 60 min. After 15 cycles, no significant reduction in the adsorption capacity was observed compared to the initial cycle. That is, the capacity has been reduced from 279.05 to 257.56 mg/g, as shown in Fig. 13. With such a number of repeated cycles, the utilization of MOF-based nanocomposites can be efficient and cost-effective for industrial adsorption purposes.

Additionally, the performance of the synthesized mix-ligand MOF (MH-20%) is compared with some reported MOF-based adsorbents in the literature in terms of the maximum adsorption capacity, isotherm, kinetic, and thermodynamic parameters. As reported in Table 9, the mix-ligand MOF nanocomposite is among the most efficient and performable adsorbents for CO_2_ capture.

**Table 9:** Performance comparison of the synthesized mix-ligand MOF (MH-20%) with other MOF-based sorbents in adsorbing CO_2_.

Sorbent	Temperature (°C)	Pressure (bar)	*q*_*eq*_ (mg/g)	*ΔH* (kJ/mol)	Best isotherm model	Best kinetic model	References
Fe(II)-MOF-74	25	0–10	24.66	–	Langmuir	–	^63^
MOF-505/GO	5–25	0–1	176.36	− 34.8	–	–	^64^
MOF UMCM-1	25–75	1	47.22	− 20	Sips	–	^65^
Amino-Zr-MOF	0–23	0–1	231.44	− 29.3	–	–	^66^
MOF-200/GO	25–75	0–1	59.37	–	Langmuir	–	^67^
Zr-MOF-808/NH_2_	25–65	1–9	369.11	− 17.36	Sips	Ritche second-order	This work

## Conclusion

Zr-BTC MOF-808 was synthesized and improved with NH_2_ ligand as a novel mixed ligand MOF-based adsorbent for CO_2_ capture. The modified adsorbent had a higher specific surface area and pore volume compared to the pure MOF-808 while maintaining similar mesoporous morphology. Three influential factors of the process; pressure, temperature, and the amount of loaded NH_2_ were studied to maximize CO_2_ adsorption capacity. The best-operating conditions were 25 °C, 9 bar, and 20 wt% NH_2_ into the MOF structure, achieving the maximum equilibrium adsorption capacity of 369.11 mg/g. The results demonstrated that the adsorption capacity directly relates to pressure and NH_2_ amount and reversely relates to temperature. The Ritchie second-order was the best-fitted kinetic model for kinetic data of CO_2_ adsorption in terms of the highest *R*^2^ (> 0.99). The Sips model fitted the experimental isotherm data related to adsorption capacity with the highest *R*^2^ (> 0.99) and the lowest AARE% (< 0.1%) values simultaneously. Additionally, the negative quantities of thermodynamic parameters, including ΔH°, ΔG°, and ΔS°, indicated that the reaction mechanism was exothermic, spontaneous with a reduced irregularity in the process. Furthermore, the prepared mix-ligand MOF adsorbent nanocomposite showed good stability due to an almost steady CO_2_ adsorption rate without any significant reduction after 15 cycles (only 8% reduction).

## Data Availability

The datasets used and/or analyzed during the current study are available from the corresponding author on reasonable request.
